# The Influence of *TLR4*, *CD14*, *OPG*, and *RANKL* Polymorphisms in Periodontitis: A Case-Control Study

**DOI:** 10.1155/2019/4029217

**Published:** 2019-06-09

**Authors:** Joana Maira Valentini Zacarias, Josiane Bazzo de Alencar, Patrícia Yumeko Tsuneto, Victor Hugo de Souza, Cléverson O. Silva, Jeane Eliete Laguila Visentainer, Ana Maria Sell

**Affiliations:** ^1^Post Graduation Program in Biosciences and Physiopathology, Department of Clinical Analysis and Biomedicine, Maringá State University, Paraná, Brazil; ^2^Department of Dentistry, Maringá State University, Paraná, Brazil; ^3^Post Graduation Program in Biosciences and Physiopathology, Basic Health Sciences Department, Maringá State University, Paraná, Brazil

## Abstract

The pathogenesis of periodontitis involves a complex interaction between the microbial challenge and the host immune response. The individual immunoinflammatory response has a great contribution in the pathogenesis of the disease and becomes a trigger in the process of bone remodeling which is a characteristic of the disease. Thus, the aim of this study was to evaluate the influence of the *TLR4* A896G (rs4986790), *TLR4* C1196T (rs4986791), *CD14* C-260T (rs2569190), *RANKL* (*TNFSF11*, rs2277438), and *OPG* (*TNFSF11B* C163T, rs3102735) polymorphisms in periodontitis. A case-control study was conducted on patients with periodontitis (*N* = 203) and controls (*N* = 213) over 30 years of age, without diabetes mellitus, acute infections, and osteoarthritis, and patients without aggressive periodontitis, i.e., stage IV and C degree of periodontitis, and any periodontal treatment performed in the last 6 months. Genotypes were determined by the PCR-RFLP and sequencing method. The frequency comparisons between case and controls were performed using the chi-square test and logistic regression (OpenEpi and SNPStats software). The risk (OR) was evaluated for values of *P* < 0.05. Differences in *TLR4*, *CD14*, *RANKL*, and *OPG* genotype and allele frequency distributions were not observed between patients and controls. However, some variants were a risk factor for the development of periodontitis when considering gender and smoking habits. The *TLR4* 896 A/G genotype was a risk factor for periodontitis in males (OR = 2.86), and the *TLR4* 1196C/C genotype was a risk factor for nonsmoking males (OR = 1.85) when compared to women. The *RANKL* A/A and the *OPG* T/C genotype was associated with the risk of the disease in nonsmoking men compared to nonsmoking women with the same genotype (OR = 1.96 and OR = 2.9, respectively). In conclusion, *TLR4*, *CD14*, *RANKL*, and *OPG* variants were not associated with periodontitis. However, *TLR4*, *RANKL*, and *OPG* polymorphisms could be a risk for periodontitis in males regardless of smoking habits.

## 1. Introduction

The pathogenesis of periodontitis involves a complex interaction between microbial challenge in the oral cavity and the immune response of the host [1]. The immune inflammatory response in periodontitis includes the innate and the acquired mechanisms that work together modulating the magnitude of the response. Activation of innate immunity mechanisms occurs as a response to initial bacterial recognition. Antigens and bacterial biofilm products, such as lipopolysaccharides (LPS), are recognized by Toll-like receptors (TLRs) expressed on the immune cell membrane surfaces, the first receptors activated during pathogen and host interaction. Another molecule that interacts with the LPS is the cluster of differentiation antigen 14 (CD14) [2]. The persistence of infection amplifies the immune response, resulting in the release of inflammatory mediators leading to damage of the connective tissue and in the process of bone resorption that occurs through the RANK (receptor activator of nuclear factor-kappaB)- RANKL-OPG (osteoprotegerin) dependent mechanism [3].

TLR2 and TLR4 have been identified as the principal receptors for bacterial cell wall components [4]. TLR4 recognizes LPS from Gram-negative bacteria. TLR2 recognizes LPS and lipoteichoic acid from Gram-positive bacteria as well as lipoproteins and peptidoglycans from both Gram-positive and Gram-negative bacteria [5, 6]. Although TLR2 and TLR4 have been shown to be important for the progression of inflammation and related bone metabolism in periodontitis [7, 8], little is known about their contributions to the disease. In TLR2, TLR4 and TLR2-TLR4 knockout mice are periodontally infected with *Porphyromonas gingivalis*; periodontal bone resorption was found as TLR4-dependent [9].

CD14 interacts with TLR4 and favors the delivery of LPS to TLR4-LBP-MD-2 complex [2]. This molecule is found as an anchored membrane protein (mCD14) and in a soluble form (sCD14). The main biological function of mCD14 is to act as a receptor to recognize and link to LPS or LPS/LBP (LPS-binding protein) complexes and mediate cell inflammatory reactions [10, 11]. The sCD14 may have an important role in potentiating the immune responses to LPS in cells lacking mCD14 surface [12]. CD14 variant was found to be associated with the severity of periodontitis [13].

Receptor activator of nuclear factor-kappaB ligand (RANKL) binds to the RANK and provides signaling for osteoclast differentiation from hematopoietic progenitor cells. The OPG is a molecule that negatively regulates the RANKL-RANK binding and inhibits bone turnover by osteoclasts [3]. The OPG molecule has three structural domains that specifically influence its biological activities [14]. This protein is a member of the TNF receptor superfamily [15]. Periodontal ligament cells can produce RANKL and OPG, and these molecules have been found in the gingival crevicular fluid of periodontitis patients [16].

As TLR4 and CD14 were previously associated with LPS hyporesponsiveness [17–19] and RANKL and OPG to the regulation of bone mass [3, 20–22], we hypothesized that the polymorphisms in the genes that codified these proteins could influence the pathogenesis of periodontitis. Thus, the aim of this study was to evaluate the influence of the polymorphisms in *TLR4* (rs4986790 and rs4986791), *CD14* (rs2569190), RANKL (*TNFSF11*, rs2277438), and OPG (*TNFSF11B*, rs3102735) in the development of periodontitis.

## 2. Material and Methods

### 2.1. Sample Selection

This case-control study was approved by the Human Research and Ethics Committee of the State University of Maringá (UEM-No. 719/2011, 2011 and 1.866.509, 2016). The studied populations were from the North and Northwest regions of the state of Paraná (22°29'30"-26°42'59"S and 48°02'24"-54°37'38"W), Southern Brazil. All the individuals voluntarily sought dental clinics at the State University of Maringá (DOD-UEM) and the Inga University Center (UNINGÁ) from January 2012 to September 2017. These participants underwent a clinical periodontal examination, and the diagnostic was confirmed by an experienced Periodontist (COS). The subjects who met all eligibility criteria of this study (patients and controls) and agreed to participate were informed about its nature and signed an informed consent form.

The selection criteria was defined according to the International Workshop for a Classification of Periodontal Diseases and Conditions of 1999 [23]. Clinical parameters were evaluated in order to classify the subjects into patients or control groups. Probing depth (PD), bleeding on probing (BOP), and clinical attachment level (CAL) were examined at six sites (mesiovestibular, vestibular, distovestibular, mesiolingual, lingual, and distolingual) of each tooth. However, it was considered only as the site with the higher clinical attachment loss in the mesial face (between mesiobuccal and mesiolingual) and distal face (between discobuccal and distolingual). In that way, for inclusion criteria, four measurements for each tooth were taken into account. The patients had to have at least 5 sites in different teeth with PD ≥5 mm, CAL ≥3 mm, and more than 25% of BOP. Moderate periodontitis was defined according to the CDC (Centers for Disease Control and Prevention) criteria as ≥2 interproximal sites with CAL ≥4 mm (not on the same tooth) and OR ≥2 interproximal sites with PD ≥5 mm (not on the same tooth). According to the new classification of periodontal diseases [24], periodontitis was defined as interdental CAL detectable at ≥2 nonadjacent teeth or buccal or oral CAL ≥3 mm with pocketing ≥3 mm detectable at ≥2 teeth. However, the observed CAL cannot be ascribed to nonperiodontitis-related causes. The control group was formed by individuals having no pocket ≥4 mm and exhibiting less than 25% of BOP. The noninclusion criteria for both groups were diabetes mellitus, acute infections, osteoarthritis, pregnancy, patients without aggressive periodontitis, i.e., stage IV and C degree of periodontitis according to the classification of periodontal diseases of 2017 [24], and those who had periodontal treatment within the last 6 months or taking antibiotics during this same time. All subjects were over 30 years of age and with at least 20 teeth in the oral cavity. Due to the great miscegenation in Brazilians, the population was classified according to a previous study by Probst et al. [25] and confirmed for our region [26]. Information on the patient's smoking history was obtained by anamnesis.

### 2.2. Blood Sample Collection and DNA Extraction

Peripheral blood samples were collected by venipuncture in a tube containing EDTA. The salting-out method [27] with some modifications [28] and the QIAamp® DNA blood mini kit (Qiagen, Valencia, CA) were used in accordance with the manufacturer's instructions to perform DNA extraction from whole blood or buffy coat. The concentration and quality of the DNA were analyzed by optical density in a Thermo Scientific NanoDrop 2000® apparatus (Wilmington, USA).

### 2.3. Genotyping of *TLR4* A896G (rs4986790) and *TLR4* C1196T (rs4986791)

*TLR4* A896G (Asp299Gly, rs4986790) and *TLR4* C1196T (Thr399Ile, rs4986791) polymorphisms were performed according to Folwaczny et al. [29] with modifications. The polymerase chain reaction (PCR) was performed in 10 *μ*L final volume with 10 ng of DNA, 1.0 ng of each primer, 3.33 mM of MgCl_2_, 0.16 mM of dNTP (Invitrogen®, Frederick, MD, USA), 1X PCR buffer (5X Green GoTaq® Flexi Buffer, Promega, USA), and 0.75 U of *Taq* Polymerase (GoTaq® DNA Polymerase, Promega, USA). Cycles were performed in the Veriti thermal cycler (Applied Biosystems): one minute at 95°C; 35 cycles of 30 seconds at 95°C, 45 seconds at 60°C (to *TLR4* A896G) or 62° (to *TLR4* C1196T), and one minute at 72°C; at the end, 10 minutes at 72°C. PCR products were digested for 10 minutes at 37°C with *Nco*I (Fermentas, Canada) for *TLR4* A896G and for 60 minutes at 37°C with *Hinf*I (Fermentas, Canada) for *TLR4* C1196T. The results were observed by electrophoresis on 3.5% agarose gel with SYBR Safe (Invitrogen Life Technologies, Grand Island, NY, USA) and visualized under UV light.

### 2.4. Genotyping of *CD14* C-260T (rs2569190)

*CD14* C-260TT (rs2569190) polymorphism was performed according to Ito et al. [30] with modifications. The PCR reaction was performed in 10 *μ*L final volume with 5 ng of DNA, 1.5 ng of each primer, 1.5 mM of MgCl_2_, 0.15 mM of dNTP (Invitrogen®, Frederick, MD, USA), 1X PCR buffer (5X Green GoTaq® Flexi Buffer, Promega, USA), and 0.5 U of *Taq* Polymerase (GoTaq® DNA Polymerase, Promega, USA). The PCR product was cleaved with *Hae*III restriction enzyme (New England Biolabs) according to the manufacturer. The results were observed through electrophoresis on 3% agarose gel with SYBR Safe (Invitrogen Life Technologies, Grand Island, NY, USA) and visualized under UV light.

### 2.5. Genotyping of *OPG* C163T (rs3102735)

The *OPG* polymorphism (*TNFRSF11B* C163T, rs3102735) was evaluated by PCR-RFLP according to Langdahl et al. [31], with modifications. The PCR reaction was performed in 10 *μ*L final volume with 5 ng of DNA, 1.2 ng of each primer, 2.0 mM of MgCl_2_, 0.12 mM of dNTP (Invitrogen®, Frederick, MD, USA), 1X PCR buffer (5X Green GoTaq® Flexi Buffer, Promega, USA), and 0.5 U of *Taq* Polymerase (GoTaq® DNA Polymerase, Promega, USA). Cycles were performed in the Veriti thermal cycler (Applied Biosystems): one minute at 95°C; 35 cycles of 30 seconds at 95°C, 45 seconds at 60°C, and one minute at 72°C; at the end, 10 minutes at 72°C. The PCR product was cleaved with using the *Vsp*I restriction enzyme (Invitrogen, USA) according to the manufacturer for three hours at 37°C. The results were observed by electrophoresis on 3% agarose gel with SYBR Safe (Invitrogen Life Technologies, Grand Island, NY, USA) and visualized under UV light. The genotyping was performed only in nonsmokers.

### 2.6. Genotyping of *RANKL* (rs2277438)

The polymorphic region of *RANKL* (*TNFSF1*, rs2277438) was amplified using the PCR with specific primers, and their sequences were determined by sequencing reaction according to Eun et al. [21] with some modifications. The PCR reaction was performed in 20 *μ*L final volume with 5 ng of DNA, 1.5 ng of each primer, 1.5 mM of MgCl_2_, 0.15 mM of dNTP (Invitrogen®, Frederick, MD, USA), 1X PCR buffer (10X buffer 50 mM Invitrogen®, USA), and 0.1 U of *Taq* Polymerase Platinum® (Invitrogen®, USA). Cycles were performed in the Veriti thermal cycler (Applied Biosystems): one minute at 95°C; 35 cycles of 30 seconds at 95°C, 45 seconds at 62°C, and one minute at 72°C; at the end, 10 minutes at 72°C. The sequencing reactions were performed with the BigDye™ terminator v3.1 cycle sequencing kit (Applied Biosystems, Thermo Fisher Scientific, USA) according to the manufacturer in an automated DNA sequencer (Applied Biosystems 3500xL). The amplified and sequenced regions were edited and evaluated using the Chromas program version 2.6.4 (https://www.technelysium.com.au) and aligned in the EMBL-EBI web program, available at https://www.ebi.ac.uk/. The nucleotide sequence used as the basis for alignment corresponding to *TNFSF11* rs2277438 is available on NCBI's website (https://www.ncbi.nlm.nih.gov/projects/SNP/snp_ref.cgi?rs=2277438). The genotyping was performed only in nonsmokers.

### 2.7. Statistical Analysis

Statistical analysis was done using SNPStats software [32] (https://www.snpstats.net/start.htm) and OpenEpi program Version 3.01 (https://www.openepi.com/Menu/OE_Menu.htm). Allele and genotype frequencies for *TLR4*, *CD14*, *RANKL*, and *OPG* were obtained, and the association between genetic polymorphisms and periodontitis was evaluated using the Chi-square test with the Yates correction and logistic regression for multivariate analyses. The Student *t*-test was used to compare the differences in age, and Fisher's exact test was used to compare the differences in gender between groups. The association tests were performed for codominant, dominant, recessive, overdominant, and log-additive genetic inheritance models, and the better inheritance model was chosen according to the minor Akaike Information Criteria (AIC) [32]. The association was determined after correcting for confounding factors using multivariate logistic regression analysis including age, gender, and smoking status covariates. Haplotype frequency estimates were carried out using the expectation-maximization algorithms. Odds ratio with 95% confidence intervals was deemed only for significant *P* values. All tests were carried out using a significance level of 5%. Genotype frequency distributions were evaluated to ensure the Hardy-Weinberg equilibrium for all genes in the populations. Quanto (http://biostats.usc.edu/software) was used to calculate the sample size from the minor allele frequency (frequency of 0.03 for *TLR4* C1196T), with a population risk of 50% and a genetic effect of 2.0, with statistical power of 80%.

## 3. Results

In this study, allele and genotype frequency distributions of *TLR4* A896G (rs4986790), *TLR4* C1196T (rs4986791), *CD14* C-260T (rs2569190), *RANKL* (rs2277438), and *OPG* C163T (rs3102735) polymorphisms were analyzed in a total of 203 patients with periodontitis and 213 subjects without the disease. Among the participants, women were 56.3%, nonsmokers were 67.3%, and the age group was between 30 and 77 years. *TLR4* and *CD14* polymorphisms were evaluated in all subjects and in nonsmoking groups. *RANKL* and *OPG* polymorphisms were analyzed only in nonsmoking groups. These groups were independently analyzed to avoid bias because smoking is a risk factor for periodontitis. Subjects were matched by gender (*P* = 0.06), and nonpairing was observed for age (*P* = 0.04). The characteristics of patients and controls are described in Table 1.

The distribution of the genotype frequencies for all analyzed genes was consistent with the Hardy-Weinberg equilibrium (*P* > 0.05). The *TLR4*, *CD14*, *RANKL*, and *OPG* genotype and allele frequency distributions are summarized in Table 2. Differences in the allele and genotype frequency distributions were not observed between patients and controls in linear analyses in the recessive, dominant, or codominant inheritance models.

After multivariate analysis (considering age and smoking habit adjustment) and stratification by gender, significant differences were observed for the TLR4, RANKL, and OPG (Table 3). In a cross-classification interaction, considering polymorphisms and gender, *TLR4* 896 A/G genotype was associated with periodontitis in men when compared to the A/A wild genotype in females (OR = 2.86, 95%CI: 1.02-8.00; all subjects), and *TLR4* 1196 C/C wild genotype and *RANKL* A/A genotype were associated with periodontitis in nonsmoking men when compared to the same genotype in nonsmoking women (OR = 1.85, 95%CI: 1.03-3.28, and OR = 1.96, 95%CI: 1.01-3.78). Still, after stratification and considering gender within polymorphism, *OPG* T/C genotype was associated with the risk of developing periodontitis in nonsmoking men compared to nonsmoking women (OR = 2.9; 95%IC: 1.03-8.14).

## 4. Discussion

The genetic factors influencing the pathogenesis of periodontal disease and single nucleotide polymorphisms (SNPs) in genes involved in the host inflammatory immune response have been studied [33–37]. Therefore, this case-control study was conducted to evaluate the possible influence of immune innate and bone resorption gene polymorphisms, *TLR4*, *CD14*, *RANKL*, and *OPG*, in periodontitis, in carefully selected patients and controls based on clinical parameters and especially in nonsmokers.

We found that these polymorphisms were not associated with the disease. However, the *TLR4*, *OPG*, and *RANKL* polymorphisms were involved in the pathogenesis of periodontitis when considering gender and smoking habits. In multivariate analyzes, nonsmoking men carrying the *TLR4* 896 A/G and 1196 C/C genotypes showed approximately 200% risk of periodontitis development compared to women carrying the wild genotypes. Added to this, those men carrying the *RANKL* A/A and *OPG* C/T genotype had 100% to 200% risk of developing the disease, compared to women carrying the same genotypes. Thus, it is possible to infer that genetic variants affecting the innate response and the process of bone resorption were factors predisposing to the disease in men, regardless of the use of cigarettes.

It is known that smokers and males are at risk for the development of periodontitis [38–46]. Smoking tobacco was an epigenetic factor and may change the transcription and methylation states of extracellular matrix organization-related genes, which exacerbate the periodontal condition [47]. In our study, smokers with periodontitis were more frequent (40.4%) than the total population of Brazilian smokers, which accounts for 17.2% [48]. Men have been considered to be more susceptible to periodontitis due to hormonal factors, personal hygiene habits, and poor health prevention habits [49]. However, our results showed that polymorphisms in immune response genes might also influence the pathogenesis of periodontitis in men, regardless of smoking habits.

As for the *TLR4* polymorphisms, our results are in concordance with others, though we found such risk only in men. In meta-analysis studies, *TLR4* 896G allele was associated with the risk of developing periodontitis [1], in a recessive model [50] and after stratification by ethnicity, only in Caucasians [13, 51]. Regarding *TLR4* 1196 C>T polymorphism, the T allele was associated with an increased risk of developing periodontitis in Caucasians in an additive model of inheritance [13]. For *CD14* polymorphisms, we did not find an association between *CD14* and periodontitis. On the other hand, a meta-analysis conducted by Han et al. [13] showed that the *CD14* -260C allele was a risk factor to the severity of periodontitis. Folwaczny et al. [52] suggested that this same allele was a risk factor for disease only in female patients. However, many reports found no association between *TLR4* and *CD14* polymorphisms and patients with periodontitis in different populations [29, 52–61].

Regarding *RANKL* polymorphism, different from that was observed in our results, no differences were observed in a study with Iranian patients with periodontitis [62] as well as in another study with adolescents with periodontitis [63]. As for *OPG* polymorphisms, studies have reported higher levels of OPG in the gingival crevicular fluid, saliva, and gingival tissues of healthy individuals than in patients with periodontitis [64–66], and Mogi et al. [66] observed higher levels of OPG in the gingival crevicular fluid in mild periodontitis compared to the moderate or severe forms. This same study also showed that the proportion of RANKL to OPG concentration in the gingival crevicular fluid was significantly higher in patients with periodontitis disease than in healthy individuals. No association study with *OPG* C163T polymorphism and periodontitis has been performed before. However, Lucena et al. [67] evaluated the influence of this polymorphism in patients with periodontitis and diabetes and no association was found. The *OPG* 163C allele was observed less frequently in this study, which corroborates with the described frequencies of this polymorphism in different populations (https://www.ncbi.nlm.nih.gov/snp/rs3102735#frequency_tab).

The exact mechanism involved in the susceptibility to periodontitis is unclear. We hypothesized that the polymorphisms in genes that codified proteins related to receptors of the immune cell membranes as well as mediators related to bone resorption were involved in periodontitis immunopathogenesis. Our results showed that *TLR4* polymorphism could be associated with disease in men. The human *TLR4* gene is located on chromosome 9q33.1 and encodes a type I transmembrane protein that has an extracellular leucine-rich repeat domain and Toll/IL-1 receptor (TIR) domain [68] that is very important for signal transduction. The *TLR4* wild-allele genotype is associated with the normal expression of the membrane protein, which is able to recognize the LPS in the complex formation of LPS-LPB-TLR4 [69–71]. A single point mutation in this domain can interfere in the response of LPS [72]. The *TLR4* A896G polymorphism results in the replacement of a conserved aspartic acid residue by a glycine at position 299; in *TLR4* C1196T, a threonine was replaced with an isoleucine in position 399. Both variants are related to the leucine-rich repeat (LRR) region, mainly in the ligand-binding (*TLR4* A896G) and in the coreceptor-binding (*TLR4* C1196T) regions [73]. The wild type has been associated with better LPS response [17].

As to bone resorption, we found that *RANKL* and *OPG* polymorphisms were a risk factor for the disease. RANKL encoded by the gene *TNFSF11* located on chromosome 13q14.11 is an anchored membrane protein expressed on osteoblasts, T and B lymphocytes, and fibroblasts in low levels; it is also cleaved in the soluble form [74]. *RANKL* (*TNFSF11* rs2277438) polymorphism is a variant in the intron 2 [21]. *RANKL* alleles were associated with susceptibility to bone involvement disease [75–77]. OPG is encoded by the *TNFRSF11B* gene located on chromosome 8q24.12. It is produced by dendritic and endothelial cells, fibroblasts, and T and B lymphocytes [20]. *OPG* polymorphism (*TNFRSF11B* C163T, rs3102735) is located in the promoter region of the gene and can influence its transcription. The C allele was associated with a lower bone mineral density or higher frequency of fractures at different bone sites [78].

The complex interaction between genetic, biological, and environmental factors increases periodontitis susceptibility, and thus, case-control studies conducted in different ethnic groups are important because some polymorphisms have differences in the frequency distributions among populations. The positive points of this study were the judicious selection of patients and controls, who had the diagnostic confirmed by sole and experienced Periodontist, and did not present predisposing factors for periodontitis-like infections and inflammatory diseases, diabetes, and bone diseases [79–84]. Age was also considered, and no one younger than 30 years old was added in the groups. Nonsmoking individuals were separately analyzed. A limitation of the study was the low statistical power obtained when analyzing subgroups, such as nonsmokers and gender. Also, the possible influence of covariate age, gender, and smoking habit on the composition of cases and controls in our sample should be considered, given their relevance in oral health and the profile of periodontitis.

## 5. Conclusion

In conclusion, *TLR4*, *CD14*, *RANKL*, and *OPG* variants were not associated with periodontitis. However, *TLR4*, *RANKL*, and *OPG* polymorphisms could be a risk for periodontitis in males regardless of smoking habits.

## Figures and Tables

**Table 1:** Characteristics of patients with periodontitis and controls.

	Patients	Controls	*P*	OR (95%CI)
*All subjects*	*N* = 203	*N* = 213		
Gender (*n* and %)^a^				
Male	99 (48.8)	83 (39.0)		
Female	104 (51.2)	130 (61.0)	0.06	
Age mean ± SD (year)^b^	47.8 ± 9.0	46.0 ± 8.8	0.04	
Smoking habit (*n* and %)^a^				
Nonsmokers	121 (59.6)	159 (74.7)		
Smokers	82 (40.4)	54 (25.3)	<0.01	2.0 (1.31-3.03)

*Nonsmokers*	*N* = 121	*N* = 159		
Gender (*n* and %)^a^				
Male	56 (46.3)	54 (34.0)		
Female	65 (53.7)	105 (66.0)	0.05	
Age mean ± SD (year)^b^	48.2 ± 9.1	46.1 ± 8.6	0.04	

*n*: number of individuals; *N*: population size; %: percentage; SD: standard deviation; *P*: *P* value; OR: odds ratio; CI: confidence interval. ^a^Comparison by Fisher's exact test; ^b^comparison by the Student *t*-test.

**Table 2:** Genotype and allele frequency distributions for *TLR4*, *CD14*, *RANKL*, and *OPG* polymorphisms in patients with periodontitis and controls (all subjects and nonsmoking individuals).

Genotypes and alleles	Nonsmokers		*P*	All subjects		*P*
	Patients	Controls		Patients	Controls	
	*N* = 100	*N* = 144		*N* = 182	*N* = 198	
	*n* (%)	*n* (%)		*n* (%)	*n* (%)	
*TLR4* A896G						
A/A	84 (84.0)	129 (90.0)		151 (83.0)	176 (89.0)	
A/G	16 (16.0)	15 (10.0)	0.28	31 (17.0)	22 (11.0)	0.13
A	184 (92.0)	273 (95.0)		333 (91.5)	374 (94.0)	
G	16 (8.0)	15 (5.0)	0.29	31 (8.5)	22 (6.0)	0.15
*TLR4* C1196T						
C/C	85 (85.0)	129 (90.0)		155 (85.2)	176 (89.0)	
C/T	15 (15.0)	15 (10.0)	0.38	27 (14.8)	22 (11.0)	0.35
C	185 (92.0)	273 (95.0)		337 (92.6)	374 (94.0)	
T	15 (8.0)	15 (5.0)	0.40	27 (7.4)	22 (6.0)	0.37
*CD14* -C260T						
C/C	29 (29.0)	39 (27.0)		48 (26.4)	55 (28.0)	
C/T	47 (47.0)	77 (53.0)	0.63	91 (50.0)	102 (52.0)	0.97
T/T	24 (24.0)	28 (19.0)	0.84	43 (23.6)	41 (21.0)	0.63
C	105 (52.0)	155 (54.0)		187 (51.4)	212 (54.0)	
T	95 (48.0)	133 (46.0)	0.85	177 (48.6)	184 (46.0)	0.62
*RANKL* rs2277438^∗^	*N* = 108	*N* = 134				
A/A	69 (63.9)	83 (61.9)		—	—	
G/A	37 (34.3)	48 (35.8)	0.89	—	—	
G/G	2 (1.8)	3 (2.3)	0.83	—	—	
A	175 (81.0)	214 (79.9)		—	—	
G	41 (19.0)	54 (20.1)	0.84	—	—	
*OPG* C163T^∗^	*N* = 111	*N* = 145				
T/T	80 (72.1)	99 (68.3)		—	—	
C/T	28 (25.2)	39 (26.9)	0.79	—	—	
C/C	3 (2.7)	7 (4.8)	0.56	—	—	
T	188 (84.7)	237 (81.7)		—	—	
C	34 (15.3)	53 (18.3)	0.45	—	—	

^∗^Polymorphism evaluated only in nonsmoking individuals. The nonpairing of the number of individuals in the studied polymorphisms occurred due to lack of some samples during the course of the study. *N*: population size; *n*: number of individuals with the allele or genotype; %: allele and genotype frequencies ×100. *P*: *P* value.

**Table 3:** *TLR4*, *RANKL*, and *OPG* genotype frequency distributions between patients with periodontitis and control after stratification according to gender.

Genotype (*n* and %)	Female			Male		*P*	OR (95%CI)
	Patients	Controls		Patients	Controls		
*All subjects* ^∗^							
*TLR4* A896G rs4986790	*N* = 223			*N* = 156			
A/A	79 (35.4)	110 (49.3)	Ref.	71 (45.5)	66 (42.3)	0.01	2.86 (1.02-8.00)
A/G	18 (8.1)	16 (7.2)		13 (8.4)	6 (3.8)		

*Nonsmokers* ^∗∗^							
*TLR4* C1196T rs4986791	*N* = 159			*N* = 85			
C/C	47 (29.6)	90 (56.6)	Ref.	38 (44.7)	39 (45.9)	0.02	1.85 (1.05-3.28)
C/T	11 (6.9)	11 (6.9)		4 (4.7)	4 (4.7)		
*RANKL* rs2277438	*N* = 144			*N* = 99			
A/A	37 (25.7)	57 (39.6)	Ref.	33 (33.3)	26 (26.3)	0.03	1.96 (1.01-3.78)
G/A	18 (12.5)	28 (19.4)		19 (19.2)	20 (20.2)		
G/G	2 (1.4)	2 (1.4)		0	1 (1.0)		
		Patients	Controls				
*OPG* C163T rs3102735		*N* = 111	*N* = 145				
T/C	Female	14 (12.6)	29 (20.0)	Ref.			
	Male	14 (12.6)	10 (6.9)			0.04	2.9 (1.03-8.14)

^∗^Multivariate analyses adjusted by age and smoking habits. ^∗∗^Multivariate analyses adjusted by age. Only significant results are shown. Ref.: genotype used as reference; *n*: number of individuals with the allele or genotype; %: allele and genotype frequencies ×100; *N*: sample size; *P*: *P* value; OR: odds ratio (adjusted values); CI: confidence interval.

## Data Availability

The genotype and haplotype data used to support the findings of this study are included within the article.
